# The concept of placebo/nocebo response and effect

**DOI:** 10.1016/j.ero.2025.09.006

**Published:** 2025-10-10

**Authors:** Fabrizio Benedetti

**Affiliations:** 1 Department of Neuroscience, University of Turin Medical School, Turin, Italy; 2 Innovative Clinical Trials, Training & Healthcare Initiative (ICTHI), Zermatt, Switzerland

## Abstract

The placebo response is different from the placebo effect. Whereas the placebo response is the global response to a placebo, including spontaneous remission, regression to the mean, patients’ and experimenters’ biases, possible effects of cointerventions, as well as psychological factors, the placebo effect includes only the psychological factors, such as the patients’ expectations and learning/conditioning. It is like the difference between drug response and drug effect: whereas the former is the global response to a drug, including all the factors described above, the latter represents the specific pharmacodynamic effect. Nocebo responses and nocebo effects go in the opposite direction; these consist of the experience of adverse events when a placebo is administered. The difference between response and effect is crucial for at least two reasons. First, clinical trials are aimed at identifying placebo responses, regardless of the mechanisms that are involved. Second, the identification of the real psychobiological placebo effect aims to understand the underlying mechanisms, that is, what is going on in the patient’s brain when he receives a placebo treatment.

## THE PLACEBO RESPONSE

A placebo response, typically observed in clinical trials, is the clinical improvement following the administration of a placebo. It includes all the factors contributing to the improvement, for example, the natural course of the disease (eg, spontaneous remissions), regression to the mean, patients’ and clinicians’ biases, symptom detection ambiguity, possible hidden effects of unidentified cointerventions, as well as patients’ and clinicians’ expectations about the therapeutic outcome [1,2]. Physicians, clinical investigators, and drug companies are mainly interested in demonstrating the superiority of a treatment compared to the placebo, regardless of the underlying mechanisms. In other words, they are not so much interested in knowing *why* an improvement occurs in the placebo group, but rather if the active treatment works better than the placebo. This pragmatic approach is typical of clinical trials, as opposed to the explanatory approach, whereby one of the main purposes is to elucidate the mechanisms underlying the symptom and/or the action of the treatment [3,4]. Most clinical trials are pragmatic, as the main interest of both physicians and drug companies is to demonstrate the efficacy of the therapy under test. By taking into account all these elements of the placebo response, in a clinical trial, there is no way to distinguish between all these factors in the absence of adequate control groups. Therefore, most (virtually all) clinical trials assess the placebo response and not the placebo effect.

## THE PLACEBO EFFECT

Although today there is still some confusion about the words ‘response’ and ‘effect’ and clinicians and clinician scientists tend to use these two words interchangeably, the real placebo effect is the psychobiological phenomenon taking place in the patient’s brain and body when he receives a placebo [1,2]. In order to investigate the real placebo effect, it is necessary to run a no-treatment group: the difference between the no-treatment group, which represents the natural history of symptom/disease, and the placebo group is the real psychobiological placebo effect [5]. Since most clinical trials do not include a no-treatment group, clinical trials do not represent a good model for studying and understanding the mechanisms underlying the placebo effect, unless the target of the trial is the understanding of the placebo effect (explanatory trial).

Today we know that there is not a single mechanism of the placebo effect and not a single placebo effect, but many [1,2]. We have to look for different mechanisms in different medical conditions and in different therapeutic interventions. Therefore, different systems and apparatuses, as well as different diseases and treatments, are affected by placebos in different ways. Expectation of a future outcome is one of the principal mechanisms [6]. In general, every procedure and condition that increases patients’ expectations can potentially boost placebo effects, ranging from high price to brand label and from the use of invasive placebos to high placebo dose (eg, many placebo pills) [1]. From a neuroscientific point of view, expecting a future event may involve several brain mechanisms aimed at preparing the body to anticipate that event. For example, the expectation of a future positive outcome may reduce anxiety and/or activate the neuronal networks of reward mechanisms [[7], [8], [9]].

Besides expectations, another important mechanism in placebo responsiveness is learning. Subjects who suffer from a painful condition and who regularly consume a medication can associate the shape, colour, and taste of a pill with a decrease in their pain. After repeated associations, a sugar pill that looks like the real medication can also decrease their pain. In place of the shape, colour, and taste of pills, several other stimuli can be associated with clinical improvement, such as syringes, stethoscopes, white coats, hospitals, doctors, nurses, and so on. The mechanism that underlies this effect is ‘conditioning’, whereby a conditioned (neutral) stimulus (like the colour or shape of a pill) can be effective in inducing the reduction of a symptom if it is repeatedly associated with an unconditioned stimulus (like the drug inside the pill) [[10], [11], [12]]. Conditioning and expectations are not mutually exclusive, as conditioning may lead to the reinforcement of expectations.

## THE NOCEBO

What has been said for the placebo also applies to nocebo, but in the opposite direction. The term nocebo (‘I shall harm’) was gradually introduced into the medical jargon in contrast to the term placebo (‘I shall please’) by some authors to distinguish the pleasing from the noxious effects of placebos [13]. Nocebo responses, or nocebo adverse events, may confound the interpretation of many clinical trials. For example, Amanzio et al [14] compared the rates of adverse events reported in the placebo arms of clinical trials for three classes of antimigraine drugs: nonsteroid anti-inflammatory drugs, triptans, and anticonvulsants. It was found that the rate of adverse events in the placebo arms of trials with antimigraine drugs was high. In addition, and most interestingly, the adverse events in the placebo arms corresponded to those of the antimigraine medication against which the placebo was compared. For example, anorexia and memory difficulties, which are typical adverse events of anticonvulsants, were present only in the placebo arm of these trials. These results suggest that the adverse events in placebo arms of clinical trials of antimigraine medications depend on the adverse events of the active medication against which the placebo is compared. These findings are certainly in keeping with the expectation theory of placebo and nocebo effects.

Much less is known today about nocebo mechanisms, mainly due to ethical constraints, because negative expectations need to be induced. Some biochemical pathways that are critical in nocebo hyperalgesia have been identified, such as cholecystokinin, cyclooxygenase, and the hypothalamus-pituitary-adrenal axis [1,2].

## CONCLUSIONS

It is important to separate the placebo response and the placebo effect, as this may have important implications in both the clinical trial setting and medical practice. A consensus on the distinction between response and effect has recently been reached, and this may lead to better doctor-patient communication [15,16]. For example, in medical practice, it would be desirable to inform patients not so much about placebo responses, but rather about placebo effects, namely, about the psychological factors that contribute to the therapeutic outcome. Likewise, doctors should be aware that better communication with their patients boosts not so much placebo responses in general but rather placebo effects, namely the psychological component. This distinction is also crucial in the setting of clinical trials because a placebo response comes from the difference between the active treatment and the placebo group. By contrast, in order to demonstrate a real psychological placebo effect, it is necessary to compare the placebo group with a no-treatment group, which is not usually done in clinical trials. Therefore, I strongly suggest using the word placebo response whenever we refer to clinical trials, whereas we should adopt the word placebo effect whenever we want to analyse how patients respond psychologically and biologically to verbal and nonverbal cues. I believe this distinction will improve both clinical research and routine clinical practice.

## Funding

This research did not receive any specific grant from funding agencies in the public, commercial, or not-for-profit sectors.

## CRediT authorship contribution statement

**Fabrizio Benedetti:** Writing – review & editing, Writing – original draft, Conceptualization.

## Competing interests

The author declares no competing interests.

## References

[1] Benedetti F. (2021).

[2] Benedetti F., Frisaldi E., Shaibani A. (2022). Thirty years of neuroscientific investigation of placebo and nocebo: the interesting, the good, and the bad. Annu Rev Pharmacol Toxicol.

[3] Benedetti F., Carlino E., Piedimonte A. (2016). Increasing uncertainty in CNS clinical trials: the role of placebo, nocebo, and Hawthorne effects. Lancet Neurol.

[4] Schwartz D., Lellouch J. (1967). Explanatory and pragmatic attitudes in therapeutical trials. J Chronic Dis.

[5] Fields H.L., Levine JD. (1984). Placebo analgesia – a role for endorphins?. Trends Neurosci.

[6] Kirsch I. (1985). Response expectancy as a determinant of experience and behavior. Am Psychol.

[7] de la Fuente-Fernández R., Ruth T.J., Sossi V., Schulzer M., Calne D.B., Stoessl AJ. (2001). Expectation and dopamine release: mechanism of the placebo effect in Parkinson’s disease. Science.

[8] Mayberg H.S., Silva J.A., Brannan S.K., Tekell J.L., Mahurin R.K., McGinnis S. (2002). The functional neuroanatomy of the placebo effect. Am J Psychiatry.

[9] Scott D.J., Stohler C.S., Egnatuk C.M., Wang H., Koeppe R.A., Zubieta JK. (2007). Individual differences in reward responding explain placebo-induced expectations and effects. Neuron.

[10] Batterman RC. (1966). Persistence of responsiveness with placebo therapy following an effective drug trial. J New Drugs.

[11] Batterman R.C., Lower WR. (1968). Placebo responsiveness – influence of previous therapy. Curr Ther Res Clin Exp.

[12] Amanzio M., Benedetti F. (1999). Neuropharmacological dissection of placebo analgesia: expectation-activated opioid systems versus conditioning-activated specific subsystems. J Neurosci.

[13] Kennedy WP. (1961). The nocebo reaction. Med World.

[14] Amanzio M., Corazzini L.L., Vase L., Benedetti F. (2009). A systematic review of adverse events in placebo groups of anti-migraine clinical trials. Pain.

[15] Evers A.W.M., Colloca L., Blease C., Annoni M., Atlas L.Y., Benedetti F. (2018). Implications of placebo and nocebo effects for clinical practice: expert consensus. Psychother Psychosom.

[16] Evers A.W.M., Colloca L., Blease C., Gaab J., Jensen K.B., Atlas L.Y. (2021). What should clinicians tell patients about placebo and nocebo effects? Practical considerations based on expert consensus. Psychother Psychosom.

